# Supplementary material for: Population Pharmacokinetics and Dosing Regimen Optimization of Latamoxef in Chinese Children
Source: Pharmaceutics. 2022 May 11;14(5):1033. doi: 10.3390/pharmaceutics14051033 (PMC9146031; doi:10.3390/pharmaceutics14051033)
Supplement: Supplementary file 1 [file pharmaceutics-14-01033-s001.zip › pharmaceutics-1679283-supplementary.pdf]

Supplemental Table S1. PTA values (%) of different dosing regimens for pediatric patients.

BSA group	Dosing regimens	MIC(μg/mL)									
		**0.25**	**0.5**	**1**	**2**	**3**	**4**	**5**	**6**	**7**	**8**
0.2-0.4m²	25mg, q12h	94.9	83.9	49.5	11.3	1.4	0.3	0	0	0	0
	50mg, q12h	99.3	94.9	83.9	49.5	23.2	11.3	5.2	1.4	0.7	0.3
	100mg, q12h	99.9	99.3	94.9	83.9	66.4	49.5	33.2	23.2	15.9	11.3
	150mg, q12h	100	99.9	98.0	92.9	83.9	72.3	60.5	49.5	38.0	29.2
	200mg, q12h	100	99.9	99.3	94.9	90.4	83.9	75.7	66.4	57.3	49.5
	250mg, q12h	100	100	99.6	96.9	93.8	89.5	83.9	76.7	69.1	62.9
	100mg, q8h	100	99.9	99.0	94.4	87.8	77.9	67.1	55.0	43.1	34.3
	150mg, q8h	100	99.9	99.8	97.3	94.4	90.7	84.9	77.9	71.0	62.2
	200mg, q8h	100	100	99.9	99.0	96.3	94.4	91.7	87.8	83.6	77.9
	100mg, q6h	100	99.9	99.9	98.1	94.5	90.7	84.7	77.2	69.7	59.4
	150mg, q6h	100	100	99.9	99.5	98.1	95.6	93.4	90.7	87.0	82.7
	175mg, q6h	100	100	99.9	99.7	98.7	96.8	94.9	92.9	90.7	87.6
	200mg, q6h	100	100	99.9	99.9	99.3	98.1	96.1	94.5	92.6	90.7
0.41-0.6m²	50mg, q12h	92.2	76.2	40.5	9.4	1.3	0.3	0	0	0	0
	100mg, q12h	97.2	92.2	76.2	40.5	18.9	9.4	4.4	1.3	0.5	0.3
	150mg, q12h	99.3	95.5	87.5	63.8	40.5	23.4	13.9	9.4	5.5	2.9
	200mg, q12h	99.7	97.2	92.2	76.2	57.7	40.5	26.6	18.9	12.9	9.4
	300mg, q12h	99.9	99.3	95.5	87.5	76.2	63.8	52.1	40.5	30.8	23.4
	350mg, q12h	99.9	99.4	96.9	89.9	80.9	69.9	59.6	50.4	40.5	32.1
	375mg, q12h	100	99.5	96.9	91.7	82.9	72.8	63.8	54.1	44.9	36.1
	450mg, q12h	100	99.9	98.0	93.7	87.5	79.7	70.8	63.8	55.3	48.2
	500mg, q12h	100	99.9	98.5	94.2	89.3	82.9	76.2	68.0	61.1	54.1
	525mg, q12h	100	99.9	98.7	94.5	89.9	84.5	77.9	69.9	63.8	56.5
	550mg, q12h	100	99.9	99.1	94.9	91.0	85.6	79.1	72.0	65.6	59.0
	200mg, q8h	99.9	99.7	97.0	91.5	82.4	71.5	58.7	47.7	36.9	27.9
	300mg, q8h	100	99.9	99.1	95.6	91.5	86.0	78.9	71.5	63.3	54.9
	350mg, q8h	100	99.9	99.5	96.0	92.6	89.2	84.2	77.6	71.5	64.4
	400mg, q8h	100	99.9	99.7	97.0	94.2	91.5	87.1	82.4	77.0	71.5
	200mg, q6h	100	99.9	99.3	95.8	92.0	87.1	79.5	71.1	61.3	53.1
	300mg, q6h	100	99.9	99.9	98.5	95.8	93.1	90.5	87.1	82.7	76.8
	325mg, q6h	100	99.9	99.9	98.8	96.4	94.2	91.5	88.7	85.2	80.8
	350mg, q6h	100	100	99.9	99.0	96.7	94.6	92.5	90.3	87.1	83.3
	400mg, q6h	100	100	99.9	99.3	97.6	95.8	94.0	92.0	90.0	87.1
	450mg, q6h	100	100	99.9	99.5	98.5	96.7	94.7	93.1	91.3	89.7
	475mg, q6h	100	100	99.9	99.5	98.7	96.9	95.5	93.7	92.3	90.5
0.61-0.8m²	100mg, q12h	92.5	78.1	48.2	13.9	4.4	1.2	0.2	0	0	0
	200mg, q12h	97.2	92.5	78.1	48.2	25.5	13.9	8.3	4.4	2.0	1.2
	300mg, q12h	99.1	95.5	88.1	67.6	48.2	31.8	21.5	13.9	10.3	6.6
	350mg, q12h	99.4	96.6	89.9	73.0	56.9	40.6	28.4	20.3	13.9	10.7
	375mg, q12h	99.4	96.9	91.5	76.3	59.9	45.1	31.8	23.0	16.5	12.0
	475mg, q12h	99.9	98.1	94.1	82.9	69.5	57.8	45.8	34.9	26.5	20.8

	550mg, q12h	99.9	98.9	94.8	86.0	75.4	63.7	53.6	43.7	34.4	27.2
	700mg, q12h	99.9	99.4	96.6	89.9	82.6	73.0	64.8	56.9	48.2	40.6
	725mg, q12h	99.9	99.4	96.7	91.1	83.3	74.7	66.6	58.5	50.6	43.0
	400mg, q8h	99.9	99.6	96.8	91.7	84.9	76.3	65.5	56.1	47.9	38.9
	500mg, q8h	100	99.8	97.9	93.7	89.0	82.9	76.3	67.8	61.3	53.0
	550mg, q8h	100	99.9	98.8	95.0	90.2	86.2	79.8	72.3	65.1	57.7
	650mg, q8h	100	99.9	99.1	95.6	92.1	88.7	84.3	79.2	72.9	66.0
	750mg, q8h	100	99.9	99.6	96.3	93.7	90.6	86.9	82.9	78.8	73.3
	400mg, q6h	100	99.9	99.3	96.1	92.5	89.0	84.6	77.4	70.8	63.0
	500mg, q6h	100	99.9	99.5	97.1	94.7	91.7	89.0	85.2	80.2	75.1
	550mg, q6h	100	99.9	99.9	97.8	95.4	92.8	90.6	87.7	83.7	79.1
	500mg, q6h(2h)	100	100	99.8	99.3	97.7	95.6	93.0	91.1	88.8	84.7
	550mg, q6h(2h)	100	100	99.9	99.5	98.0	96.2	94.2	92.3	90.5	87.9
	575mg, q6h(2h)	100	100	99.9	99.5	98.4	96.7	94.6	92.5	91.0	89.0
	600mg, q6h(2h)	100	100	99.9	99.6	98.6	96.9	95.1	93.0	91.1	89.5
	625mg, q6h(2h)	100	100	99.9	99.6	98.7	97.3	95.6	93.5	91.8	90.3
0.81-	200mg, q12h	93.9	83.2	60.6	25.9	11.3	4.9	1.8	0.7	0.2	0.1
1.0m^2	300mg, q12h	96.4	90.2	75.3	46.7	25.9	14.3	8.5	4.9	2.6	1.4
	450mg, q12h	98.6	94.4	85.7	65.1	46.7	31.8	21.7	14.3	10.6	7.3
	550mg, q12h	99.2	95.6	91.0	71.8	56.7	42.8	30.5	21.9	16.1	11.7
	750mg, q12h	99.8	97.8	93.6	81.8	68.7	57.8	46.7	36.9	28.7	23.3
	850mg, q12h	99.9	98.4	94.2	85.0	73.2	62.6	53.2	43.6	35.2	28.5
	500mg, q8h	99.9	98.8	95.5	87.5	78.0	66.3	55.7	46.2	36.0	28.4
	600mg, q8h	99.9	99.4	95.9	90.0	82.8	74.2	64.1	55.7	48.1	39.2
	750mg, q8h	99.9	99.7	97.1	92.6	87.5	81.3	74.2	66.3	59.3	52.3
	825mg, q8h	99.9	99.7	97.7	93.5	88.8	83.9	77.8	70.8	63.9	57.2
	900mg, q8h	100	99.9	98.3	94.9	90.0	86.2	80.5	74.2	67.7	62.0
	600mg, q6h	99.9	99.9	98.9	95.1	91.6	88.4	83.0	77.0	70.6	63.3
	700mg, q6h	100	99.9	99.3	96.3	93.3	90.2	86.9	82.3	77.0	71.0
	600mg, q6h(2h)	100	99.9	99.7	98.0	95.4	92.8	90.2	86.8	82.2	75.1
	700mg, q6h(2h)	100	99.9	99.7	98.6	96.5	94.0	91.9	90.0	86.8	82.7
	800mg, q6h(2h)	100	99.9	99.8	99.1	97.4	95.4	93.0	91.6	89.7	86.8
	850mg, q6h(2h)	100	100	99.8	99.2	97.7	95.8	93.7	91.9	90.4	87.9
	925mg, q6h(2h)	100	100	99.9	99.5	98.1	96.4	94.5	93.0	91.5	89.9
	950mg, q6h(2h)	100	100	99.9	99.5	98.2	96.7	94.8	93.0	91.7	90.1
1.01-	300mg, q12h	93.8	83.0	61.9	29.6	13.6	7.3	3.3	1.4	0.5	0.2
1.2m^2	350mg, q12h	94.3	85.8	67.7	37.0	18.8	10.6	5.7	2.7	1.4	0.6
	500mg, q12h	96.7	91.8	78.7	54.1	34.0	21.5	13.6	9.0	6.0	3.7
	750mg, q12h	98.8	95.2	87.7	69.6	54.1	41.1	29.6	21.5	15.5	11.5
	900mg, q12h	99.3	96.2	89.9	75.5	61.9	49.6	38.7	29.6	22.6	17.7
	925mg, q12h	99.4	96.3	90.5	76.4	62.3	50.5	40.3	31.0	23.8	18.5
	1100mg, q12h	99.6	97.3	93.2	81.4	68.9	58.8	48.2	39.9	31.6	25.2
	800mg, q8h	99.9	98.8	95.4	88.2	80.6	71.5	61.7	53.7	45.1	37.4
	900mg, q8h	99.9	99.2	95.9	90.0	83.2	75.5	66.5	58.5	51.4	44.3

1000mg, q8h	99.9	99.5	96.5	91.4	85.9	78.9	71.5	63.5	57.0	50.3
1200mg, q8h	99.9	99.6	97.3	92.7	88.2	83.2	77.7	71.5	65.0	58.5
1400mg, q8h	100	99.9	98.2	94.9	90.2	86.6	81.5	76.8	71.5	65.7
900mg, q6h	99.9	99.9	98.8	94.9	91.7	88.4	84.2	78.9	73.0	66.9
1000mg, q6h	100	100	99.1	95.7	93.0	89.6	86.3	81.9	77.4	72.0
850mg, q6h(2h)	100	99.9	99.7	97.7	94.8	92.5	89.9	86.9	82.5	76.8
900mg, q6h(2h)	100	99.9	99.7	98.0	95.3	93.1	91.2	88.1	84.6	79.5
950mg, q6h(2h)	100	99.9	99.7	98.2	95.8	93.2	91.6	89.2	86.0	81.9
1000mg, q6h(2h)	100	99.9	99.7	98.3	96.0	93.7	91.8	89.9	87.0	83.3
1050mg, q6h(2h)	100	99.9	99.7	98.6	96.4	94.1	92.3	90.6	88.1	85.1
1150mg, q6h(2h)	100	99.9	99.7	98.8	97.2	95.0	93.1	91.7	89.7	87.2
1200mg, q6h(2h)	100	99.9	99.7	98.9	97.4	95.3	93.2	91.8	90.2	88.1
1300mg, q6h(2h)	100	99.9	99.8	99.1	97.8	95.9	94.0	92.7	91.2	89.6
1350mg, q6h(2h)	100	100	99.8	99.3	98.0	96.0	94.4	93.1	91.7	89.9
1400mg, q6h(2h)	100	100	99.9	99.3	98.1	96.4	94.7	93.1	91.8	90.6

PTA, probability of target attainment; BSA, body surface area; MIC, minimum inhibitory concentration.
